# Beyond sutures: a focused review of contemporary concepts in periodontal wound closure

**DOI:** 10.2340/biid.v13.47084

**Published:** 2026-10-27

**Authors:** Mohammed S. AlQranei

**Affiliations:** Department of Preventive Dental Sciences, College of Dentistry, Imam Abdulrahman Bin Faisal University, Dammam, Saudi Arabia

**Keywords:** Periodontics, sutures, tissue adhesives, wound healing

## Abstract

Wound closure is a critical part of periodontal and mucogingival surgery, as it directly affects tissue stability, healing, patient comfort, and esthetic outcomes. This focused review examines the current evidence on periodontal wound closure, with particular attention to suture materials, microsurgical suturing, tissue adhesives, and suture removal protocols. The available literature suggests that closure materials differ in their biologic and mechanical behavior, with monofilament and synthetic sutures generally demonstrating less bacterial retention and lower tissue reactivity than braided materials. Microsurgical suturing appears to offer advantages in soft tissue adaptation and esthetic refinement, although supporting evidence remains limited in certain regenerative procedures. Tissue adhesives, particularly cyanoacrylate-based formulations, may provide a useful alternative in selected cases, with studies reporting satisfactory healing and improved patient comfort, although the evidence remains limited. The studies also suggest that suture removal should be based on the specific surgical procedure and healing requirements rather than on a routine time-based rule. Overall, the evidence remains heterogeneous and often limited, supporting thoughtful material selection and procedure-specific clinical judgment rather than definitive recommendations.

**KEY MESSAGES**

- Microsurgical suturing may improve selected clinical and patient-centered outcomes, although supporting evidence remains limited. Tissue adhesives may also provide comparable healing and enhanced patient comfort in selected cases, but the available evidence remains heterogeneous and limited.
- Antibacterial-coated sutures could reduce early bacterial colonization, but their clinical benefit remains inconsistent, making them more suitable for selected high-risk cases than routine use.
- Suture removal should be individualized according to the procedure and healing demands, as the conventional 7-day approach is not universally supported by evidence.

## Introduction

Surgical interventions are a fundamental component of periodontal therapy, ranging from resective procedures to advanced regenerative techniques. While considerable attention has been directed toward flap design, biomaterials, and surgical outcomes, the role of sutures and related wound-closure approaches remains comparatively underexplored in the periodontal literature [1]. Sutures are not only essential for wound closure but also play an integral role in stabilizing tissues, maintaining hemostasis, and facilitating optimal healing [2].

The choice of suture material and wound-closure approach can significantly influence surgical outcomes, including flap adaptation, graft containment, postoperative inflammation, and patient comfort. Moreover, inappropriate suture selection or placement may contribute to complications such as dehiscence, infection, or delayed healing. As periodontal procedures become increasingly refined, particularly with the rise of microsurgery and minimally invasive approaches, a more nuanced understanding of suture dynamics is warranted [3].

Despite the central role sutures play in surgical success, current clinical practice is often guided more by personal preference than by standardized protocols or high-quality evidence. Variations in wound closure approach, suture material characteristics, and removal timing can lead to inconsistent outcomes. There is a growing need for a focused evaluation of these variables to establish evidence-based recommendations that can improve healing predictability, enhance patient comfort, and reduce postoperative complications in periodontal surgery. Accordingly, this focused mini-review aimed to critically summarize selected contemporary evidence regarding suture materials, antibacterial-coated sutures, microsurgical suturing, suture-removal timing, and tissue adhesives in periodontal wound closure, with emphasis on their biologic behavior, clinical performance, and practical implications.

## Methods

A focused literature search was initially conducted on 9 February 2026 using PubMed/MEDLINE and Google Scholar, covering literature published through January 2026, with no lower date restriction. The search used combinations of terms related to periodontal wound closure, including periodontal surgery, suture materials, absorbable and non-absorbable sutures, monofilament and braided sutures, antibacterial-coated sutures, periodontal microsurgery, microsurgical suturing, tissue adhesives, fibrin sealant, fibrin adhesive, cyanoacrylate, and suture removal. Additional searches were conducted during manuscript preparation and revision. To improve reproducibility, supplementary searches were conducted on 24 September 2026, retaining the original publication cutoff of 31 January 2026. The complete supplementary PubMed strategy, Google Scholar queries, and retrieval and screening details are provided in Supplementary Search Strategy S1. Reference lists of relevant articles and reviews were also examined to identify additional studies. The author screened titles and abstracts for relevance and subsequently assessed the full texts of potentially eligible articles. Articles were included when they addressed wound-closure materials or techniques relevant to periodontal, mucogingival, implant, or closely related oral surgical procedures and reported clinical, microbiological, histological, healing, or patient-reported outcomes. Articles that did not address wound-closure materials or techniques within this scope, or did not report relevant outcomes, were excluded.

Original clinical, microbiological, and histological studies were given particular emphasis when supporting specific clinical observations, while systematic reviews, meta-analyses, and other relevant reviews were used to place individual findings within the broader body of evidence. As this article was intended as a focused narrative mini-review rather than a systematic review, formal duplicate screening, quantitative synthesis, and study-level risk-of-bias assessment were not performed. Instead, the literature was considered qualitatively, with greater weight given to studies that were methodologically sound, directly relevant to periodontal or oral surgical wound closure, consistent with the wider evidence, and clinically applicable.

## Discussion

### Suture material characteristics: absorbability, filament structure, composition, and surface properties

Suture materials differ in several clinically relevant characteristics, including absorbability, filament structure, chemical composition, natural or synthetic origin, and surface properties. Together, these features affect handling, duration of wound support, microbial adherence, and local tissue response. Absorbability largely determines how long a material remains within the tissues and whether postoperative removal is required. Absorbable sutures such as chromic gut and polyglactin progressively lose tensile strength as they degrade, whereas non-absorbable materials such as nylon, polypropylene, and polytetrafluoroethylene (PTFE) remain in place until removed and can provide prolonged mechanical support when needed [4]. Tissue response also varies between materials, with composition and degradation behavior influencing the degree of local inflammation during healing [5].

Filament structure and surface characteristics are particularly relevant in the oral environment, where sutures are continuously exposed to saliva, plaque, and a diverse microbiota. Braided sutures generally provide favorable handling and knot security, but their interstitial spaces may facilitate microbial accumulation. Monofilament materials have smoother surfaces and fewer niches for bacterial retention; however, their greater memory and lower surface friction may make handling more technique-sensitive [5]. In a study of 50 patients following periodontal flap surgery, higher bacterial counts were reported on non-absorbable than absorbable sutures 1 week postoperatively, with mean colony-forming units of 59.8 and 22.9, respectively [6]. Although this study grouped the materials according to absorbability, other studies comparing specific suture types provide additional insight into the role of filament structure and material characteristics. In an in vitro study, Katz et al. found that braided silk harbored approximately five to eight times more bacteria than nylon, which showed the lowest bacterial adherence among the sutures evaluated [7]. Similarly, a randomized dentoalveolar surgery study of 60 patients reported comparatively lower microbial adherence on the absorbable monofilament poliglecaprone 25 (Monocryl) [8]. These findings highlight the relevance of filament architecture and surface characteristics when selecting sutures for the plaque-rich oral environment.

Material composition is also reflected in tissue response. In a periodontal study comparing braided silk with expanded polytetrafluoroethylene (ePTFE), silk produced a more pronounced inflammatory reaction in the surrounding gingival tissues [9]. This is consistent with broader evidence showing greater tissue reactivity with silk compared with several synthetic alternatives [5]. Additional oral-surgery evidence comparing silk with polyethylene terephthalate has also reported differences in postoperative tissue response [10]. More recently, PTFE sutures demonstrated significantly lower aerobic and anaerobic biofilm accumulation than silk following third-molar surgery [11]. Although these findings are derived from a non-periodontal surgical setting, they provide useful supportive evidence regarding material behavior in the oral environment.

Overall, suture selection in periodontal surgery should balance the biological and mechanical properties of the material with the demands of the procedure. Absorbability, filament structure, surface characteristics, and composition all contribute to clinical performance (Table 1). Selection should therefore consider the surgical site, required duration of stabilization, plaque-control requirements, handling characteristics, and planned follow-up. Operator experience and preference are also important considerations in suture selection and should not be overlooked in clinical practice. A survey of Turkish periodontists found a strong preference for non-absorbable monofilament sutures in periodontal and implant surgery, further highlighting the influence of clinical familiarity and individual preference on material selection [12].

**Table 1:** Summary of comparison between different conventional sutures.

Suture material	Absorbability	Filament structure	Composition/origin	Surface/coating	Main clinical consideration
Chromic gut	Absorbable	Monofilament	Natural collagen	Chromium-treated [4, 5]	Resorbable; relatively greater tissue reactivity [4, 5]
Polyglactin 910 (Vicryl)	Absorbable	Braided	Synthetic copolymer	Coated, triclosan-coated formulation available [4, 13, 14]	Good handling; potential microbial retention [4, 5]
Poliglecaprone 25 (Monocryl)	Absorbable	Monofilament	Synthetic copolymer	Smooth surface [4]	Low microbial adherence; shorter-term support [4, 8]
Silk	Non-absorbable	Braided	Natural protein fiber	Braided/coated surface [4, 5]	Good handling; higher bacterial retention and tissue reaction [5, 7, 9, 11]
Nylon	Non-absorbable	Monofilament	Synthetic polyamide	Smooth surface [4, 7]	Low bacterial adherence and tissue reactivity [4, 5, 7]
Polypropylene	Non-absorbable	Monofilament	Synthetic polyolefin	Smooth surface [4, 5]	Low tissue reactivity; prolonged support [4, 5]
PTFE/ePTFE	Non-absorbable	Structure varies by formulation	Synthetic fluoropolymer	Low-adhesion fluoropolymer surface [4, 9, 11]	Prolonged support; favorable tissue and microbial profile [4, 9, 11]

PTFE: polytetrafluoroethylene; ePTFE: expanded polytetrafluoroethylene.

### Antibacterial or coated sutures in periodontal surgery: do they improve outcomes?

The use of antibacterial or coated sutures in oral and implant surgery has received increasing attention, yet clinical consensus remains elusive. A comprehensive review by Makrygiannis et al. assessed various coated sutures, particularly triclosan and chlorhexidine, highlighting their capacity to reduce early bacterial colonization without consistently improving clinical outcomes [13]. In a randomized clinical trial by Tabrizi et al., polyglactin 910 coated with triclosan (Vicryl Plus) was compared with its non-coated counterpart during dental implant surgery, demonstrating no significant difference in healing or complication rates [14]. Similarly, a recent scoping review by La Rosa et al. investigated findings from five clinical studies and concluded that antibacterial sutures do exhibit antimicrobial activity; however, the clinical impact on wound healing and infection prevention in oral surgery was limited and inconsistent [15]. A broader meta-analysis by Suleiman et al., which encompassed surgical disciplines beyond dentistry, found that antibiotic-coated sutures significantly reduced surgical site infections, suggesting potential cross-applicability to oral surgery contexts [16]. Etemadi et al. reported that similar bacterial counts accumulated on triclosan-coated sutures compared to silk sutures after dental implant placement, and both groups showed substantial colonization by day 7 [17]. More directly relevant, a recent randomized controlled trial by Chaganti et al. comparing triclosan- and chlorhexidine-coated sutures to uncoated ones revealed lower bacterial colonization in coated groups, in addition to better healing indices and less postoperative pain, with triclosan showing the most pronounced effect [18].

Taken together, these studies suggest an antibacterial benefit of coated sutures in the early postoperative period, but with limited translation into superior clinical outcomes. While coated sutures may offer adjunctive value in high-risk cases, their routine use in periodontal and implant surgery should be guided by individual patient risk profiles and surgical complexity.

### Microsurgical suturing techniques in periodontics: clinical benefits and limitations

Finer sutures, particularly those of 6-0 or 7-0 gauge, have become a cornerstone of periodontal microsurgery, especially in procedures involving papilla preservation and root coverage. Their reduced diameter allows for atraumatic soft tissue handling, precise approximation of wound edges, and improved flap adaptation, all essential in esthetically sensitive zones. When used under magnification, such as with microsurgical loupes or an operating microscope, these sutures facilitate tension-free closure and contribute to primary intention healing, minimizing flap distortion and promoting vascular preservation [19]. Burkhardt and Hürzeler described the use of microsurgical techniques and fine sutures to improve precision in periodontal plastic surgery [19]. Jindal et al., in a small, randomized split-mouth study involving seven patients and 30 recession sites, reported comparable root-coverage outcomes with some favorable clinical measures in the microsurgical group; however, the small sample limits the certainty of these findings [20]. Despite the growing popularity of microsurgical techniques in periodontics, there remains a noticeable lack of literature specifically evaluating the tensile strength and long-term performance of fine sutures (6-0 and smaller) in regenerative procedures. Moreover, the widespread adoption of fine suturing techniques is often constrained by practical barriers, including the need for advanced training, increased surgical time, and access to specialized magnification and microsurgical instruments, all of which may discourage their use in everyday clinical settings.

More broadly, wound-closure requirements in clinical practice vary according to the surgical procedure, flap design, and need for tissue stabilization. A summary of procedure-specific wound-closure considerations is presented in Table 2.

**Table 2:** Summary of procedure-specific wound closure considerations in periodontal surgery.

Procedure	Main closure/stabilization objective	Commonly used approach and clinical consideration
Access flap surgery	Stable re-approximation of flap margins with minimal tension	Simple interrupted sutures are commonly used for flap approximation; sling sutures may be utilized when the flap must be stabilized around the teeth or at a different vertical position. Technique should follow flap design and desired final position [2, 3]
Regenerative surgery	Maintain stable, tension-free primary closure and protect the regenerative wound	Papilla-preservation and minimally invasive approaches favor primary closure. Mattress sutures, with additional interrupted sutures when needed, can provide stable interdental adaptation and reduce tension across the wound [21–23]
Root-coverage procedures	Maintain coronal flap position and close adaptation to the recipient bed	Sling sutures, alone or combined with simple interrupted sutures, are commonly used to stabilize coronally advanced flaps. Available evidence does not establish a single superior suturing protocol [24, 25]
Free gingival graft	Immobilize the graft and maintain close contact with the vascular recipient bed	Interrupted sutures, with or without periosteal fixation, are commonly used for graft stabilization. Sling and other stabilizing approaches are also described; current evidence does not clearly favor one technique [26]
Connective tissue graft	Stabilize the graft and maintain the position of the overlying flap	Sling sutures are frequently combined with interrupted or mattress sutures, depending on whether a coronally advanced or tunnel approach is used. Technique selection is mainly determined by flap design and the required graft stability [26]
Implant surgery	Stable, tension-free adaptation of peri-implant soft tissues	Simple interrupted sutures are commonly used for routine flap closure. In esthetic immediate implant procedures, sling sutures may also be used around the provisional restoration to stabilize the coronally positioned flap. Closure should be adapted to the flap design and whether augmentation is performed [3, 27, 28]

### When to remove sutures in periodontal surgery: is the 7-day rule evidence-based?

The traditional recommendation to remove intraoral sutures at approximately 7 days is largely derived from general surgical teaching rather than procedure-specific evidence in periodontics. Experimental data suggest that the timing of suture removal can influence early wound healing. In a controlled animal study, Parirokh et al. compared suture removal at 3, 5, and 7 days after raising and repositioning a mucoperiosteal flap in rabbits and evaluated healing using clinical inflammation and gingival indices. Removal at 5 days resulted in more favorable soft-tissue conditions than removal at either 3 or 7 days, leading the authors to conclude that 5 days represented the most advantageous interval in that model [29]. Nevertheless, as these findings were derived from an animal model, their direct applicability to clinical periodontal surgery is limited.

More specific evidence is available for periodontal plastic surgery, particularly coronally advanced flap (CAF) procedures. In a systematic review and meta-analysis, Tatakis and Chambrone investigated the effect of suturing protocols on root-coverage outcomes in Miller Class I/II recession defects treated with CAF. They compared randomized clinical trials with ‘early’ (<10 days) versus ‘late’ (≥10 days) suture removal and found a significantly higher proportion of sites achieving complete root coverage when sutures were removed at or after 10 days, whereas the type of suture material (absorbable vs. non-absorbable) had no influence when both were left in place for ≥10 days [24]. However, removal timing was not itself randomized, and the comparison was based on differences in protocols across studies; therefore, other procedural factors may have influenced the observed association. Complementing these findings, Pippi’s clinical review on post-surgical soft-tissue wound monitoring in periodontal and implant surgery emphasizes that flap stability and intimate adaptation to the underlying root or bone surface are critical during the first 10–14 days, particularly in coronally advanced and regenerative procedures, and that sutures should not be removed solely based on an arbitrary time point [27].

In practice, the timing of suture removal in periodontal surgery should be individualized, balancing biological requirements with patient comfort. For simple access or resective flap procedures in non-esthetic areas, removal around 5–7 days may be reasonable once adequate flap stability and healing are evident. For CAF, tunnel techniques, and regenerative procedures, retention for approximately 10 days, and in selected cases up to 14 days, may be considered when prolonged stabilization is clinically warranted [24, 27]. These intervals should be viewed as pragmatic, procedure-specific considerations rather than established rules. To reduce plaque accumulation and irritation when longer retention is needed, monofilament, low–plaque-retentive materials (e.g. PTFE or polypropylene) may be preferable to braided sutures [4, 27, 30]. In cases where individual sutures become loose or bothersome, selective removal or trimming may improve comfort while preserving key stabilizing sutures. Clear preoperative communication with patients regarding the possibility of longer suture retention in advanced soft-tissue and regenerative procedures can help align expectations and improve adherence to postoperative care.

### Tissue adhesives in mucogingival surgery

The application of tissue adhesives in mucogingival procedures presents several practical advantages over conventional suturing techniques. By eliminating the use of needles, these materials reduce the risk of accidental needlestick injuries to both surgeons and surgical assistants [31]. Depending on their formulation and indication, tissue adhesives can assist in wound approximation and maintain closure during the early phases of healing. Some formulations may also reduce microbial contamination and provide favorable esthetic outcomes. Tissue adhesives may be considered in clinical situations where rapid wound approximation with minimal tissue manipulation is preferred, such as periodontal flap closure, free gingival graft stabilization, and tissue graft donor-site management [32].

Among the tissue adhesives investigated in periodontal surgery, fibrin sealants and cyanoacrylate-based adhesives represent distinct material classes with different mechanisms and clinical roles. Fibrin sealants primarily support hemostasis and tissue stabilization through biologic clot formation. Pulikkotil et al. reported lower gingival crevicular fluid levels of the inflammatory mediators IL-1β and IL-8 following the use of a fibrin sealant, suggesting a potentially favorable early biologic response [33]. Another clinical study also suggested that fibrin sealant may provide effective flap stabilization with rapid hemostasis and reduced early postoperative plaque accumulation and discomfort compared with silk sutures [31]. However, a systematic review of four randomized clinical trials involving 101 patients found the overall certainty of evidence to be very low because of methodological limitations and risk of bias, supporting a cautious interpretation of fibrin sealants as an adjunct rather than an established replacement for sutures in periodontal surgery [34].

Cyanoacrylate esters, including N-butyl and isoamyl 2-cyanoacrylate, have been more widely evaluated as alternative wound-closure materials in periodontal surgery. These adhesives polymerize rapidly upon contact with moisture and oral fluids, forming a protective film over the wound surface [35]. Their biologic response appears to vary during healing. An early histologic investigation reported localized granulomatous reactions followed by fibroblast infiltration after cyanoacrylate application [35]. In clinical and histological comparisons with silk sutures, N-butyl cyanoacrylate was associated with reduced inflammatory cell infiltration during early healing and favorable connective tissue organization [36, 37]. Studies evaluating isoamyl 2-cyanoacrylate have similarly reported satisfactory wound approximation, hemostasis, and clinical healing following periodontal flap surgery [38–40].

Patient-reported outcomes have also been evaluated, particularly with cyanoacrylate formulations. Several clinical studies reported less postoperative pain or discomfort compared with conventional silk sutures [38–41], with some studies also noting favorable esthetic perception related to the absence of visible sutures [38, 39, 41]. A recent systematic review and meta-analysis of tissue adhesives in periodontal flap surgery found overall healing outcomes comparable with conventional suturing [42]. However, the available studies involve different adhesive formulations and are generally limited in size and methodology; therefore, these findings should be interpreted according to the specific material evaluated rather than generalized to all tissue adhesives. Overall, fibrin sealants appear to function mainly as biologic and hemostatic adjuncts, whereas cyanoacrylate formulations may serve as an alternative method of wound approximation in selected periodontal applications. A summary of the tissue adhesives discussed is presented in Table 3.

**Table 3:** Summary of comparison between different tissue adhesives.

Adhesive type	Mechanism	Reported clinical findings	Clinical role	Key limitation
Fibrin sealant	Biologic fibrin clot formation	Hemostasis, stabilization, possible favorable early inflammatory response [31, 33, 34]	Mainly adjunctive	Evidence certainty very low
N-butyl cyanoacrylate	Rapid moisture-activated polymerization	Favorable early healing versus silk in small clinical/histologic studies [35–37]	Alternative wound approximation in selected low-tension sites	Limited studies; possible local inflammatory/foreign-body response
Isoamyl 2-cyanoacrylate	Rapid polymerization forming protective surface film	Satisfactory healing, hemostasis, and patient-reported outcomes [38–41]	Alternative or adjunct in selected periodontal wounds	Small heterogeneous clinical studies

## Conclusion

Wound closure is a critical determinant of periodontal surgical success, influencing tissue stability, healing dynamics, patient comfort, and esthetic outcomes. Current evidence suggests that no single closure material or method is universally superior and selection should be tailored to the biologic and mechanical demands of the procedure. Monofilament and synthetic sutures generally show favorable biologic behavior, while tissue adhesives may be useful in selected applications. Nevertheless, the available evidence is heterogeneous, and several findings are derived from small studies or indirect evidence from non-periodontal settings. Therefore, clinical recommendations should be interpreted as evidence-informed considerations rather than definitive protocols, with wound-closure and suture-removal decisions individualized according to procedure-specific requirements.

## Supplementary Material



## Data Availability

Data sharing is not applicable to this article, as no new data were created or analyzed in this study.
