# Supplementary material for: Beyond sutures: a focused review of contemporary concepts in periodontal wound closure
Source: Biomater Investig Dent. 2026 Oct 27;13:47084. doi: 10.2340/biid.v13.47084 (PMC13720940; doi:10.2340/biid.v13.47084)
Supplement: Supplementary file 1 [file BIiD-13-47084-s1.pdf]

Supplementary material has been published as submitted. It has not been copyedited or typeset by Biomaterial Investigations in Dentistry.

Supplementary Search Strategy S1

Search dates and scope

The initial literature search was conducted on 9 February 2026, with additional searches during manuscript preparation and revision. The supplementary searches documented below were conducted on 24 September 2026 to improve reproducibility while retaining the original publication cutoff of 31 January 2026. The reported queries and counts describe these supplementary searches.

PubMed

Search date: 24 September 2026.

Complete search strategy:

```
(
"Periodontal Diseases"[Mesh]
OR "Periodontics"[Mesh]
OR "Oral Surgical Procedures"[Mesh]
OR periodont*[tiab]
OR gingiv*[tiab]
OR mucogingiv*[tiab]
OR oral[tiab]
OR dental[tiab]
OR dentoalveolar[tiab]
)
AND
(
"Sutures"[Mesh]
OR "Suture Techniques"[Mesh]
OR "Tissue Adhesives"[Mesh]
OR sutur*[tiab]
OR microsurg*[tiab]
OR "tissue adhesive*"[tiab]
OR cyanoacrylate*[tiab]
OR "fibrin sealant*"[tiab]
OR "fibrin adhesive*"[tiab]
OR "fibrin glue"[tiab]
)
AND
("1000/01/01"[dp] : "2026/01/31"[dp])
```

No additional filters were applied. The search retrieved 7,523 records. Screening was limited to the first 500 results in PubMed’s Best Match order; titles were examined, with abstracts reviewed for potentially relevant records.

Google Scholar

Search date: 24 September 2026.

The following queries were searched separately:

1. periodontal suture materials
2. periodontal antibacterial sutures
3. periodontal microsurgical suturing
4. periodontal suture removal
5. periodontal tissue adhesives
6. periodontal fibrin sealant
7. periodontal cyanoacrylate

Results were ranked by relevance. The first five pages for each query were screened by title and available summary, corresponding to 50 results per query and 350 result entries overall. Publication dates of potentially eligible articles were checked against the cutoff of 31 January 2026. The screening count includes repeat appearances of articles across queries and does not represent unique publications.
